# Development and Application of Unmanned Aerial High-Resolution Convex Grating Dispersion Hyperspectral Imager

**DOI:** 10.3390/s24175812

**Published:** 2024-09-07

**Authors:** Qingsheng Xue, Xinyu Gao, Fengqin Lu, Jun Ma, Junhong Song, Jinfeng Xu

**Affiliations:** 1 College of Physics and Optoelectronic Engineering, Department of Information Science and Engineering, Ocean University of China, Qingdao 266100, China; 2 Laboratory for Regional Oceanography and Numerical Modeling, Qingdao Marine Science and Technology Center, Qingdao 266200, China; 3 Engineering Research Center of Advanced Marine Physical Instruments and Equipment, Ministry of Education, Qingdao 266100, China; 4 Basic Teaching Center, Ocean University of China, Qingdao 266100, China

**Keywords:** optical design, hyperspectral imager, convex grating, hyperspectral remote sensing, image classification

## Abstract

This study presents the design and development of a high-resolution convex grating dispersion hyperspectral imaging system tailored for unmanned aerial vehicle (UAV) remote sensing applications. The system operates within a spectral range of 400 to 1000 nm, encompassing over 150 channels, and achieves an average spectral resolution of less than 4 nm. It features a field of view of 30°, a focal length of 20 mm, a compact volume of only 200 mm × 167 mm × 78 mm, and a total weight of less than 1.5 kg. Based on the design specifications, the system was meticulously adjusted, calibrated, and tested. Additionally, custom software for the hyperspectral system was independently developed to facilitate functions such as control parameter adjustments, real-time display, and data preprocessing of the hyperspectral camera. Subsequently, the prototype was integrated onto a drone for remote sensing observations of *Spartina alterniflora* at Yangkou Beach in Shouguang City, Shandong Province. Various algorithms were employed for data classification and comparison, with support vector machine (SVM) and neural network algorithms demonstrating superior classification accuracy. The experimental results indicate that the UAV-based hyperspectral imaging system exhibits high imaging quality, minimal distortion, excellent resolution, an expansive camera field of view, a broad detection range, high experimental efficiency, and remarkable capabilities for remote sensing detection.

## 1. Introduction

Hyperspectral remote sensing technology is a robust and comprehensive interdisciplinary tool characterized by real-time data acquisition, richness in spectral information, cost effectiveness, extensive coverage, as well as high-resolution capabilities that facilitate mapping [1]. Its applications span various domains within earth sciences, establishing it as an effective technological instrument for geological mapping [2], mining exploration [3], vegetation surveys [4], oceanic remote sensing [5], agricultural monitoring [6], atmospheric studies [7], and environmental surveillance [8], among others, thereby playing an increasingly vital role in these fields. In 2019, Yuye Huang et al. conducted precise classifications of different water qualities by investigating the apparent characteristics of water bodies in the Pearl River Estuary and analyzing their categories [9]. In 2021, Zaiming Zhou et al. utilized drone-derived spectral features to identify mangrove communities, *Spartina alterniflora*, and other plant species, performing detailed classification analyses [10]. In 2023, Junichi Kurihara et al. applied unmanned airborne hyperspectral remote sensing technology to model and predict rice yields across varying growth environments, achieving high predictive accuracy [11]. Furthermore, in 2024, Hongcheng Liu et al. employed a self-developed unmanned airborne full-spectrum hyperspectral imager for hyperspectral data acquisition aimed at identifying lithology, minerals, and other mineralization elements in uranium and beryllium deposits, resulting in successful application outcomes [12].

In recent years, unmanned aerial vehicle (UAV) hyperspectral remote sensing technology has emerged as a prominent area of research within the field of hyperspectral imaging. By integrating a stabilizing gimbal and advanced data processing software, this technology enables real-time observation of various objects within a localized area, facilitating the acquisition of spectral information and the stable output of “spectral image” data. The inherent advantages of UAVs, including high flexibility and low operational costs, render them significantly valuable for monitoring the dynamic proliferation of *Spartina alterniflora* [13]. However, the quality of data and detection efficiency in UAV-based hyperspectral remote sensing are influenced by several parameters, such as the field of view angle, spectral resolution, and the mass and volume of the mounted hyperspectral camera. Consequently, achieving a hyperspectral imaging system that features a large field of view, high spatial resolution, optimal spectral resolution, compact size, lightweight construction, and enhanced real-time performance has become a critical goal in the design of UAV-mounted hyperspectral imaging systems.

In this paper, we present the design and development of a high-resolution convex grating dispersive hyperspectral imaging system based on the Offner configuration. This system boasts several advantages, including a large relative aperture, an extended slit, excellent dispersive linearity, high spectral resolution, compact size, lightweight construction, and minimal spectral bending and chromatic aberration. Additionally, we developed a software system for the upper computer that facilitates control of the hyperspectral imager, enabling parameter adjustments, data collection, processing, and real-time data display. Both ground scanning tests and aerial scanning experiments were conducted, demonstrating high imaging quality. The experimental data were systematically categorized and evaluated [14,15].

## 2. Design of an Unmanned Airborne Hyperspectral Imager System

### 2.1. Optical System Design

The optical system of the hyperspectral imager is comprised of a front telescope system and a spectral imaging system. In accordance with the requirements for unmanned airborne applications involving large field-of-view and high-resolution hyperspectral imaging detection, the primary technical specifications of the hyperspectral imager are presented in Table 1.

**Figure 1:**
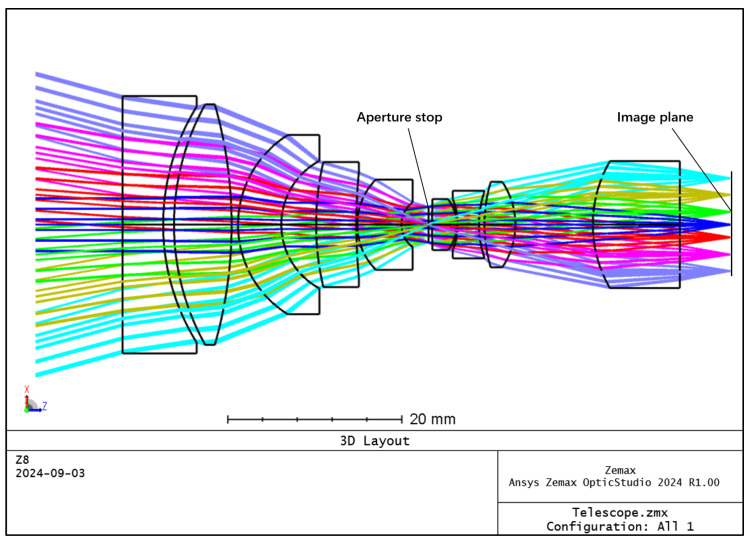
Optical structure of the front telescope system.

**Figure 2:**
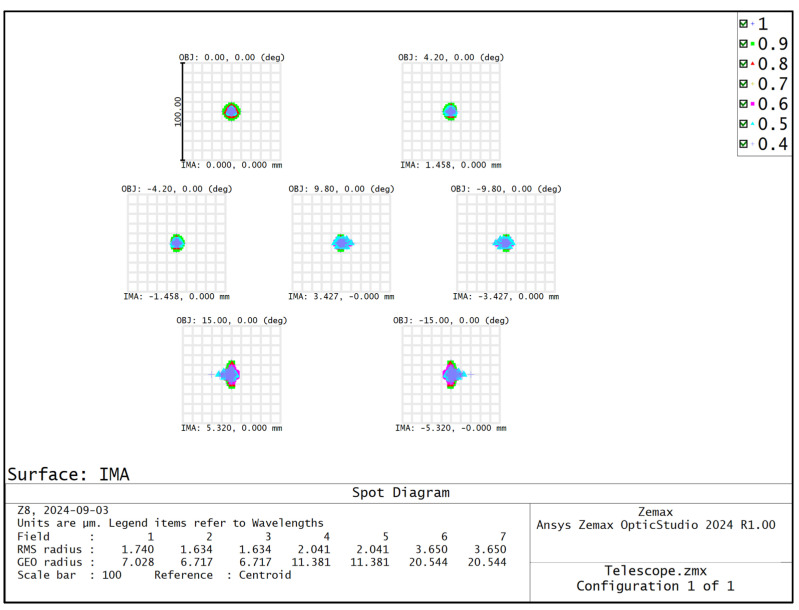
Distribution of point columns on the image plane of the telescope system.

**Figure 3:**
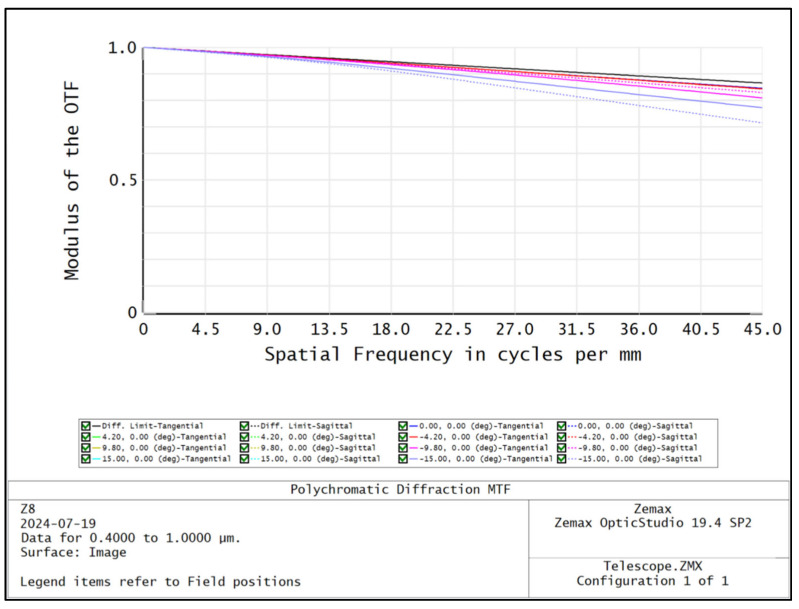
Optical transfer function curve of the front telescope system.

The spatial resolution of the hyperspectral imager is primarily determined by the telescope system. The front telescope system features a field of view of 30° and a relative aperture of 1/3, providing both a wide field of view and a significant relative aperture. To achieve optical pupil matching, the front telescope system employs an image-square telecentric optical path, while the spectral imaging system utilizes an object-square telecentric structure. The front telescope system is designed on the basis of the double Gaussian objective lens structure for complexity, and the structure of the front telescope system is shown in Figure 1. The distribution of the dot pattern on the image plane of the front telescope system is shown in Figure 2, and the radius of the RMS value of the dot pattern in the full field of view is less than 3.7 μm. The modulated optical transfer function curve of the front telescope system is shown in Figure 3, and the MTF is more than 0.8@45 lp/mm, which obtains good imaging quality in the full field of view at the same time.

**Figure 4:**
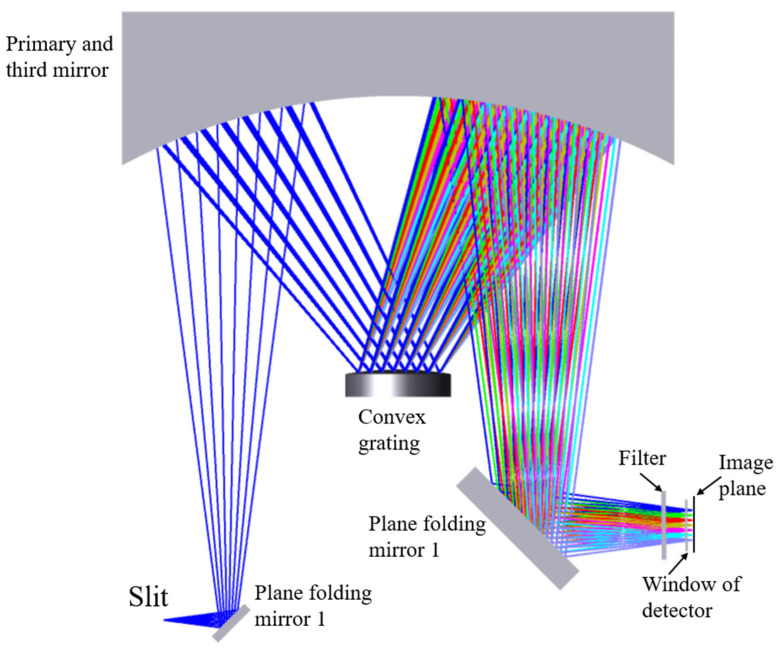
Optical structure of Offner spectral imaging system.

The spectral imaging system features a slit length of 11 mm and a numerical aperture of 0.17, characterized by a large relative aperture and an elongated slit. In contrast to interferometric spectral imaging methods, the dispersive spectral imaging approach allows for intuitive spectrum acquisition without necessitating complex computational processing. Additionally, compared to prism-based dispersion spectral imaging, grating dispersion spectral imaging offers improved dispersion linearity and higher spectral resolution. The Offner spectral imaging system based on convex grating dispersion is a new type of spectral imaging system developed on the basis of the reflective Offner relay optical system, with two spherical mirrors as the primary mirror and three mirrors and a convex grating as the second surface. In the initial structure of the Offner relay optical system, the primary mirror and the three mirrors have the same radius of curvature and center of curvature and the aperture diaphragm in the secondary mirror, so the system’s entry and exit pupils are located at infinity, and the system can be regarded as symmetric about the diaphragm system; the coma and aberrations are eliminated due to the symmetry. When the second surface is replaced by a grating, although the symmetry of the system is destroyed to a certain extent, the detector angle can be adjusted appropriately to obtain good imaging quality in a wide band at the same time. Compared with the conventional method of using gratings in collimated beams, the Offner spectral imaging system has the advantages of small size, light weight, and low spectral bending and color distortion. In order to further reduce the size and weight of the whole system, planar folding mirrors 1 and 2 are incorporated in the incident and exit arms, respectively. The Offner spectral imaging system is an off-axis optical system, and the main mirror and the three mirrors share the same spherical mirror in order to facilitate the mounting and adjusting. The optical structure of the Offner spectral imaging system is shown in Figure 4, with spectral dimensions in the paper plane and spatial dimensions in the direction of perpendicular to the paper plane. The band range of the spectral imaging system is 400–1000 nm, and negative filters are added in front of the detector window in order to avoid grating-level interference.

**Figure 5:**
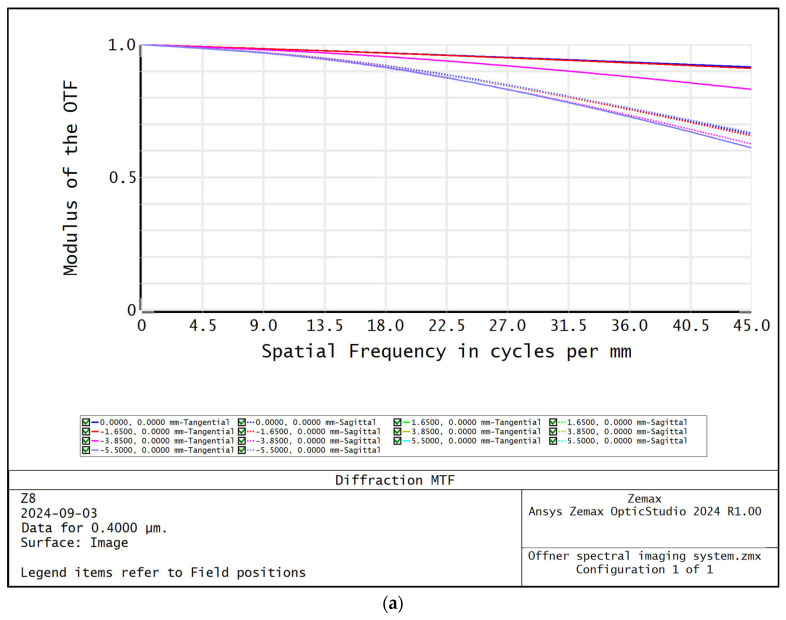

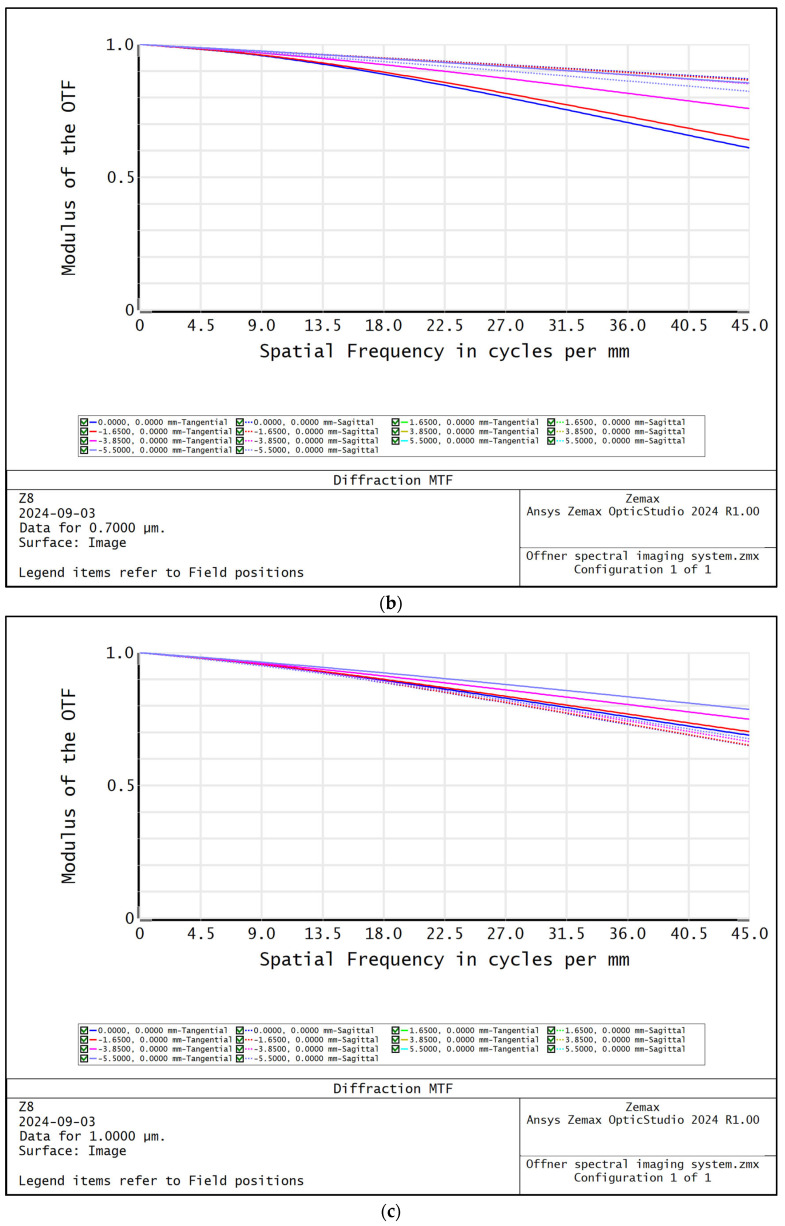
MTF curves for different wavelengths of Offner spectral imaging system: (**a**) 400 nm; (**b**) 700 nm; (**c**) 1000 nm.

**Figure 6:**
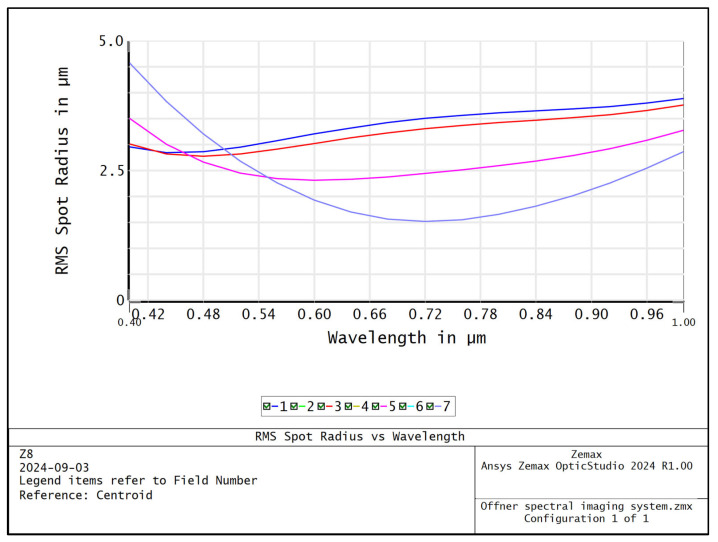
Curve of RMS radius versus wavelength for Offner spectral imaging system.

**Figure 7:**
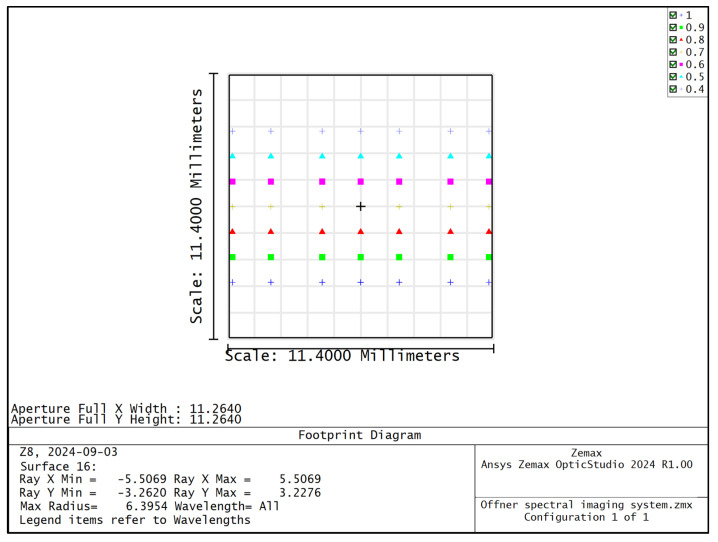
Map of light imprints on the image plane of Offner spectral imaging system.

The MTF curves of different wavelengths of the Offner spectral imaging system are shown in Figure 5, and the MTFs at the center and edge wavelengths are more than 0.6@45 lp/mm. Figure 6 shows the relationship between the radius of RMS value of the dot-plot and the wavelength, and the RMS values of the dot-plot of the different fields of view in the range of 400–1000 nm are all less than 4.6 μm, which means that the surface spectral imaging system obtains good imaging quality within the range of a wide range of wavelengths. The surface spectral imaging system obtains good imaging quality in a wide range of wavelengths, which meets the design index requirements of the spectral imaging system. Figure 7 shows the light imprint on the image surface of the spectral imaging system, the dispersion range of the 400–1000 nm band on the image surface is 6.466 mm, the width of the slit *S* is 22 μm, and the diameter of the diffuse spot of the dot-row diagram *D* is 9.2 μm; then, the design spectral resolution (FWHM) Δ*λ* of the Offner spectral imaging system can be expressed as follows:

(1) $$\Delta \lambda =\frac{d\lambda }{dl}\cdot \sqrt{S^{2}+D^{2}}$$

where *dλ*/*dl* is the reciprocal of the line dispersion rate, and the design spectral resolution (FWHM) of the Offner spectral imaging system is calculated to be 2.6 μm, which meets the index requirement of a spectral resolution better than 5 nm.

**Figure 8:**
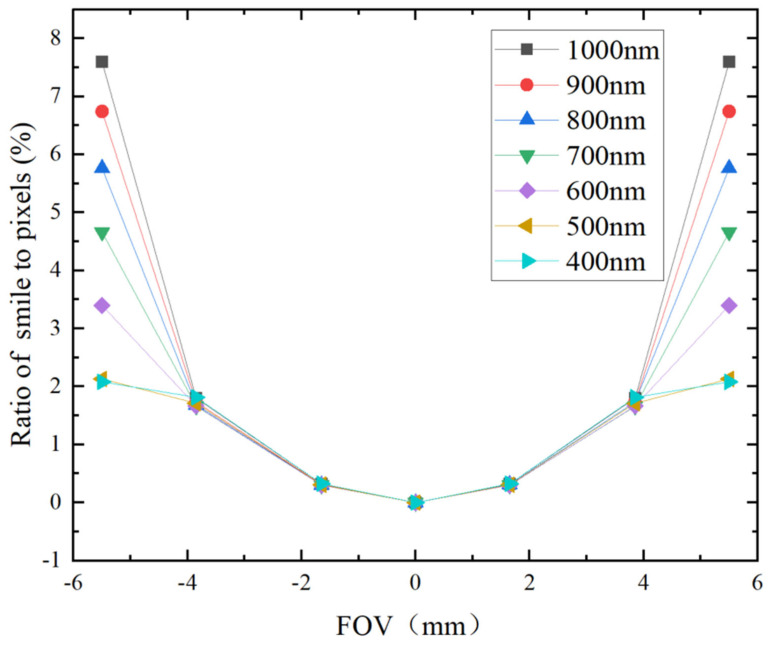
Spectral bending at different wavelengths of Offner spectral imaging system.

**Figure 9:**
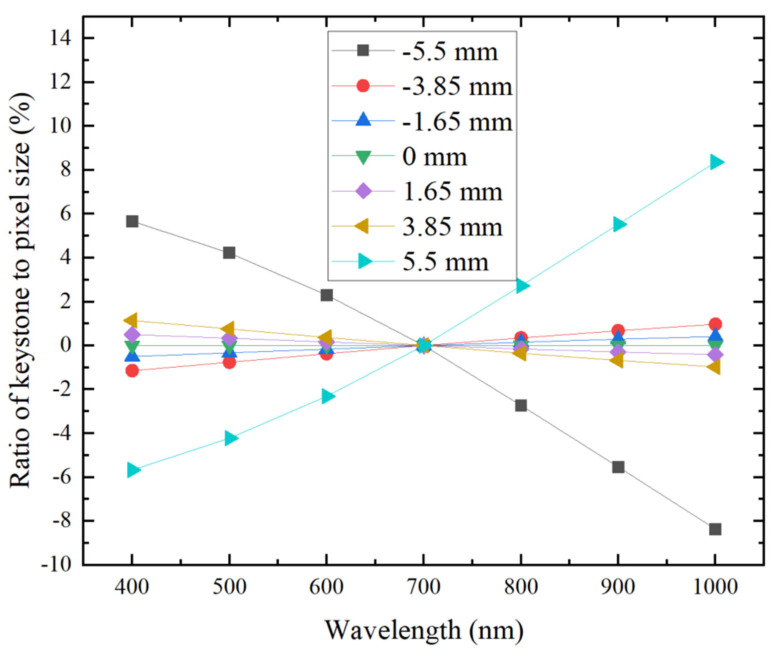
Spectral band bending for different fields of view of Offner spectral imaging system.

Spectral bending and spectral band bending are critical technical indicators for dispersive spectral imaging systems. Spectral bending refers to the extent of deviation of the slit-bending image for different wavelengths from a straight line, while spectral band bending indicates the deviation of image points corresponding to various wavelengths from a straight line that is perpendicular to the slit, as formed by the same point within the field of view on the incident slit. The spectral line bending for different wavelengths in the Offner spectral imaging system is illustrated in Figure 8, while Figure 9 depicts the spectral band bending across various fields of view. As shown in Figure 8, the degree of spectral line bending is symmetric with respect to the center of the field of view and increases with increasing wavelength, with the maximum spectral line bending measuring only 7.6% of one image element size. Figure 9 further demonstrates that as the field of view expands, the amount of spectral band bending also increases, with the maximum spectral band bending remaining below 8.4% of one image element size. From these analytical results, it is evident that the Offner spectral imaging system achieves high imaging quality across its entire operational bandwidth, thereby satisfying the imaging quality requirements expected of a spectral imaging system.

**Figure 10:**
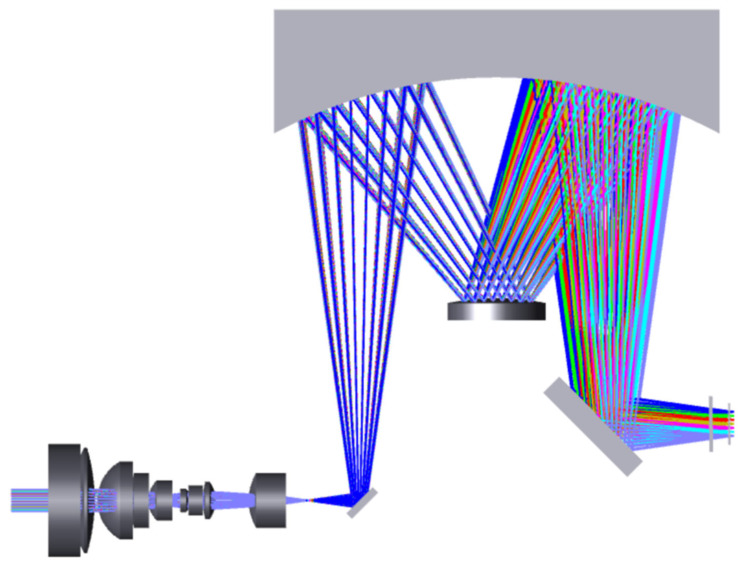
System-wide optical structure of the hyperspectral imager.

**Figure 11:**
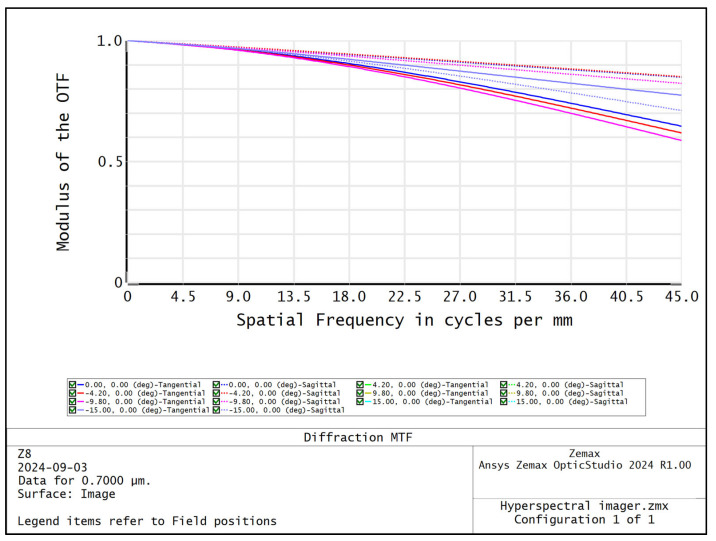
System-wide optical transfer function curve of hyperspectral imager.

**Figure 12:**
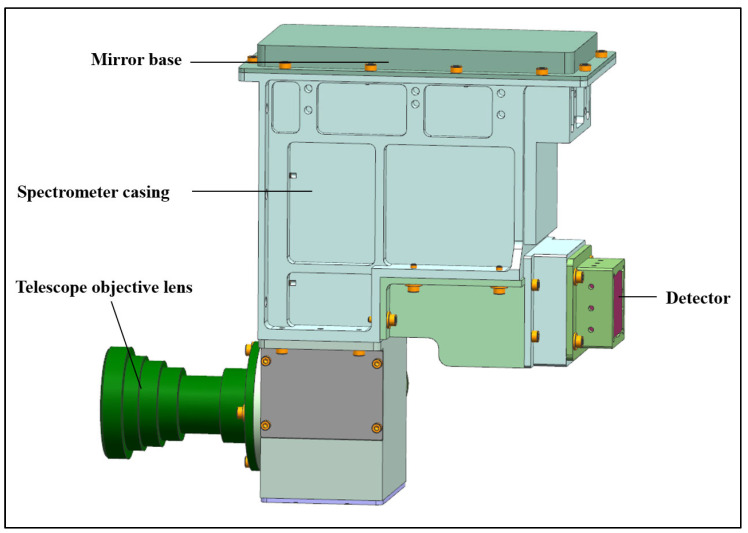
Overall mechanical structure of the hyperspectral prototype.

The previously designed image square telecentric front telescope system is connected with the Offner spectral imaging system to obtain a large field of view high-resolution hyperspectral imager full system; the optical structure is shown in Figure 10, and the volume is only 186 mm × 147 mm × 58 mm, which has the characteristics of miniaturization and light weight and is suitable for unmanned aerial hyperspectral remote sensing applications. Compared with the advanced Nano HP airborne hyperspectral imaging system from Headwall on the current market, the prototype developed in this paper has a larger field of view, a wider detection range, more spatial pixels, and higher spatial resolution at the same focal length. Figure 11 shows the optical transfer function curve of the hyperspectral imager whole system, and the MTF of the hyperspectral imager whole system is more than 0.58@45 lp/mm, which meets the imaging quality requirements of the hyperspectral imager. The mechanical structure adapted to the optical system is designed according to the overall design results of the optical system, and the design results are shown in Figure 12.

### 2.2. Spectral Calibration

Spectral calibration and testing are essential components in the development of hyperspectral imagers, as the accuracy of spectral calibration directly impacts the validity of the acquired spectral data. The observational data obtained from the hyperspectral imager can only be accurately interpreted following spectral calibration, which establishes the relationship between the theoretical center wavelength, pixel position, and the spectral bandwidth of the hyperspectral imaging system. This calibration process is crucial for determining the precise locations for subsequent data shearing and splicing of monochrome images across different wavelength channels. Ultimately, this lays the groundwork for the development of the shearing and splicing algorithms in the subsequent stages of software development.

**Figure 13:**
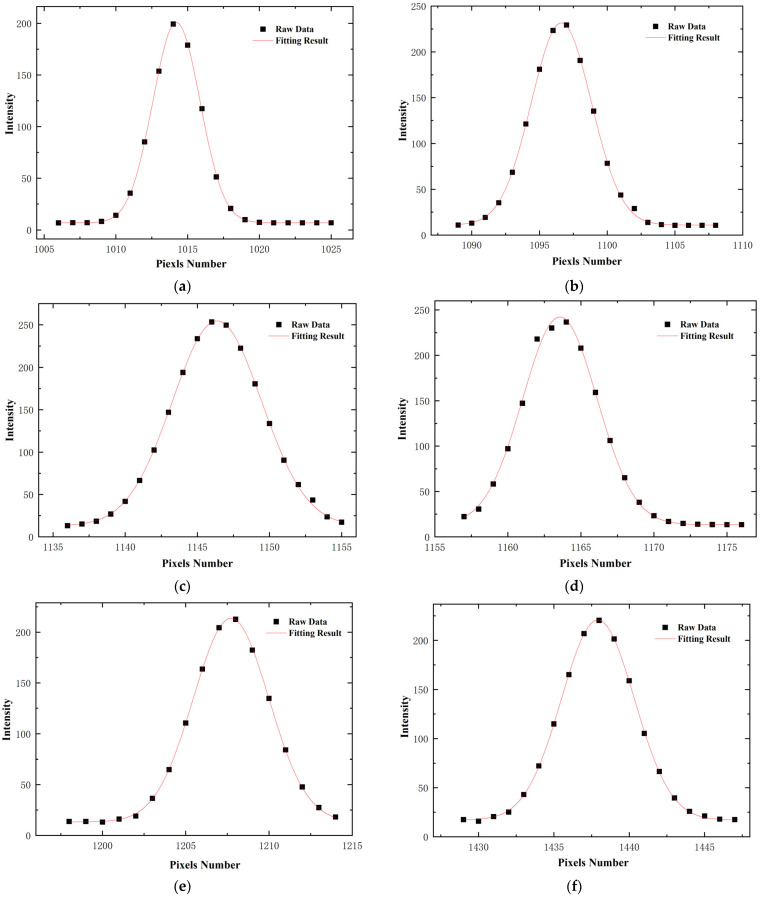
Results of Gaussian fitting of some characteristic peaks: (**a**) 696.54 nm wavelength characteristic peak fitting result; (**b**) 738.40 nm wavelength characteristic peak fitting result; (**c**) 763.51 nm wavelength characteristic peak fitting result; (**d**) 772.40 nm wavelength characteristic peak fitting result; (**e**) 794.82 nm wavelength characteristic peak fitting result; (**f**) 912.30 nm wavelength characteristic peak fitting result.

**Figure 14:**
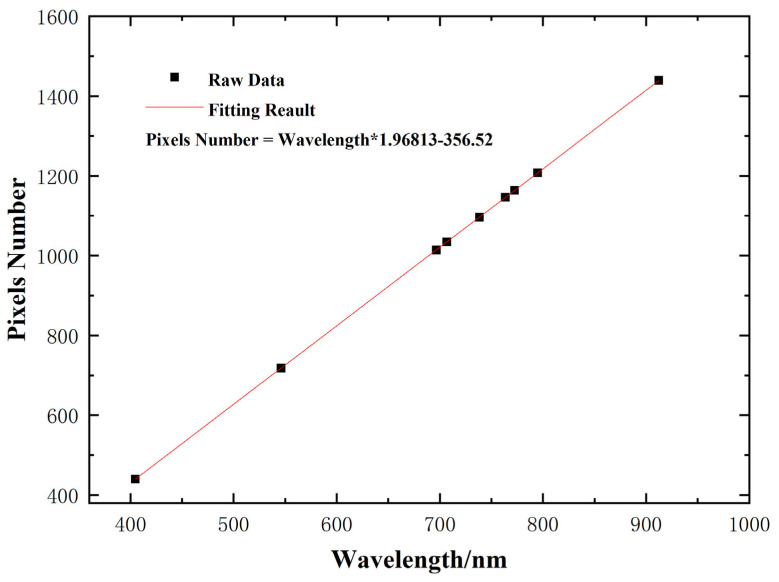
Hg lamp calibration fitting results.

Since a mercury lamp has the advantages of narrow emission spectral lines, high intensity, stable operation, and known characteristic wavelengths, a mercury lamp can be used as a spectral calibration light source. After sufficient preheating of the mercury lamp, it is placed directly in front of the slit of the spectrometer to irradiate the hyperspectral imaging instrument and obtain the original data image of the mercury lamp spectral lines on the detector image plane of the hyperspectral imaging instrument. Because the image resolution is 2048 × 2048, and the spectral line curvature of the hyperspectral imaging system developed with this paper is very small, using MATLAB R2023a to read the 1024th line in the middle of the image for calibration can well represent the linear relationship between wavelength and pixel. We next extract data from several characteristic wavelengths, including 404.66 nm, 546.08 nm, 696.54 nm, 706.72 nm, 738.40 nm, 763.51 nm, 772.40 nm, 794.82 nm, and 912.30 nm, for Gaussian fitting. Partial fitting results are shown in Figure 13, which can obtain the center pixel position of the characteristic wavelength and calculate the full width at half maximum (FWHM). Then, each feature wavelength is linearly fitted with the center pixel position obtained by Gaussian fitting, and the linear correspondence between pixel values and the center pixel position is obtained. The linear fitting results are shown in Figure 14, and the spectral resolution calculation results are shown in Table 2.

### 2.3. Software System Development

In conjunction with the development of the hyperspectral instrument, software for the hyperspectral camera was also developed using the Visual Studio platform and the C# programming language. This software enhances the usability of the camera during subsequent experimental processes.

**Figure 15:**
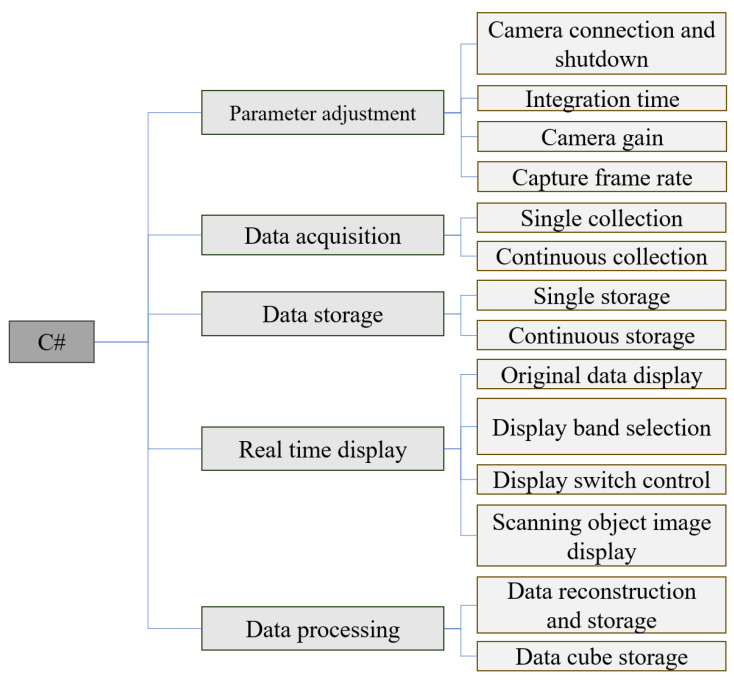
Overall functional block diagram of hyperspectral control software system.

The software system adopts a modular and integrated design concept, and five modules including instrument parameter tuning, data acquisition, data storage, real-time display, and data processing were designed and developed according to the functional requirements of the software, as shown in Figure 15. The five modules are independent of each other and can be called through the set interface. The instrument tuning module sends tuning instructions by calling the interface in the detector SDK to set the camera integration time, camera gain, and acquisition frame rate. The data acquisition module controls the camera acquisition mode and preprocesses the collected raw spectral data into images, which are then transmitted to the interface for real-time display. The data storage module stores the collected raw spectral data in the selected data format. In the real-time display module, one can select the desired frequency band to display. The built-in algorithm of the module will process and concatenate the scanned object image of the selected frequency band and display it in real-time on the interface. The data processing module cuts and splices the collected and stored raw spectral data to achieve image reconstruction, outputting monochrome images in different bands, and can also further output cube data in ENVI standard format.

**Figure 16:**
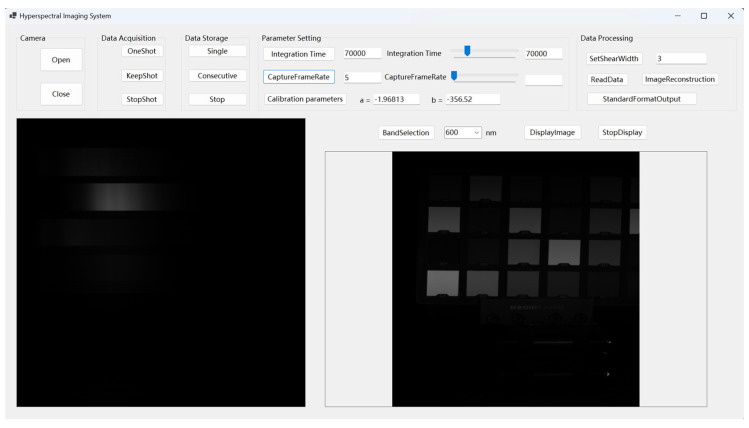
Screenshot of Software System Operation Test.

The main design interface is depicted in Figure 16. The upper section comprises the functional button area for each module, while the lower section consists of two image display areas. The first display presents the collected raw spectrum to facilitate focusing, and the second displays the real-time visualization of the reconstructed object image based on the selected spectral data. This arrangement allows for a more effective assessment of both the quality of the acquired data and the progress of the scanning experiment. During the experimental process, it is imperative to perform data acquisition, data display, data storage, and real-time visualization of single-band object images simultaneously. To achieve this, the software design of the system employs multi-threaded programming. This methodology prevents prolonged CPU occupation by individual tasks, thereby significantly enhancing the efficiency of hyperspectral imaging.

### 2.4. Outdoor Large-Range Rotary Scanning Test Experiment

**Figure 17:**
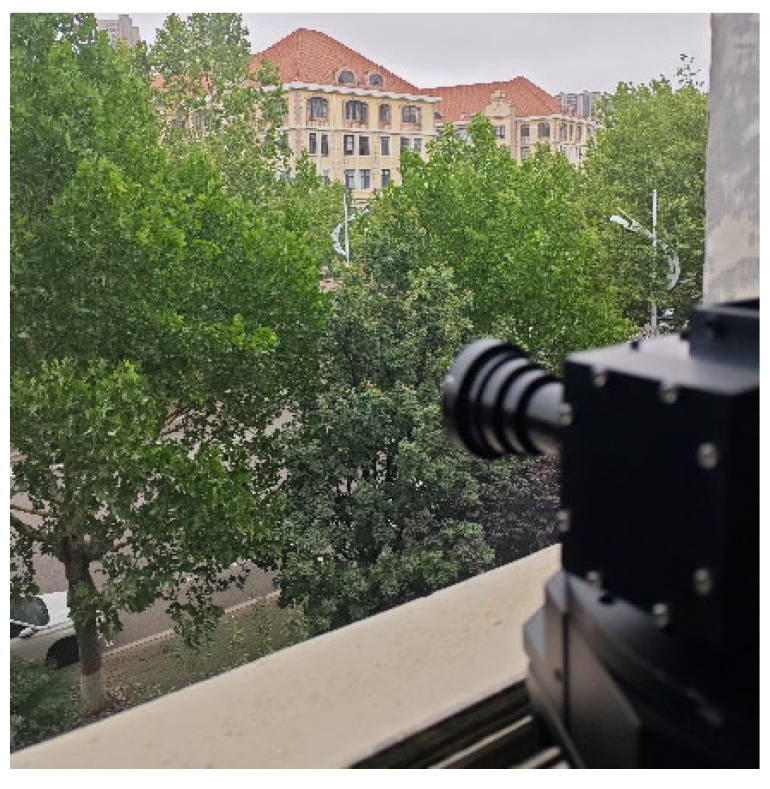
Hyperspectral Imager System Outdoor Rotary Scanning Experiment.

**Figure 18:**
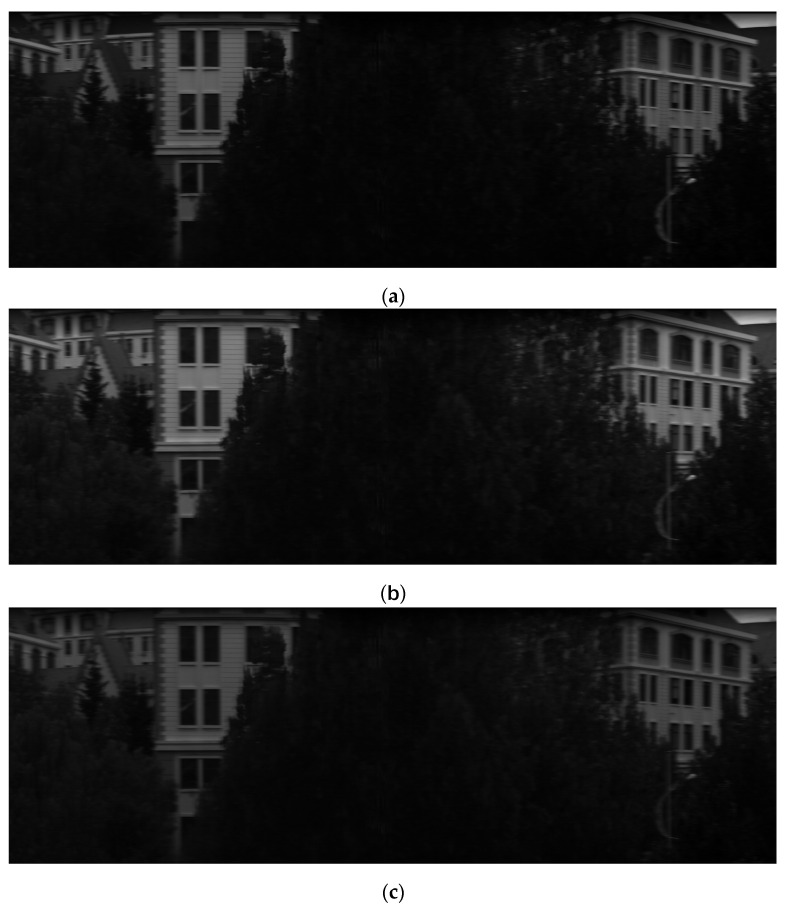

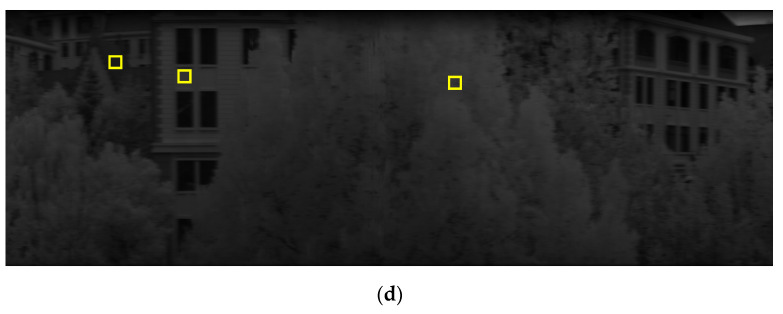
Monochromatic images at different wavelength bands from the outdoor push-scan of the hyperspectral imaging system: (**a**) monochromatic image in the 500 nm wavelength band; (**b**) monochromatic image in the 600 nm wavelength band; (**c**) monochromatic image in the 700 nm wavelength band; (**d**) monochromatic image in the 800 nm wavelength band.

Following the completion of the study on the high-resolution convex grating dispersive hyperspectral imager system, the system prototype underwent performance testing experiments. Under clear weather conditions, the hyperspectral prototype was mounted on a rotary displacement stage positioned in a window, as depicted in Figure 17. The rotational speed and direction of the stage were configured, and the developed control software was utilized to adjust the parameters of the hyperspectral camera. The rotary stage was then activated to commence data storage, continuing until the maximum displacement was reached, at which point data storage was halted. The data processing module integrated within the software subsequently analyzed the collected raw data, generating monochromatic images for various wavelengths. Specifically, monochromatic diagrams corresponding to wavelengths of 500 nm, 600 nm, 700 nm, and 800 nm are presented in Figure 18.

**Figure 19:**
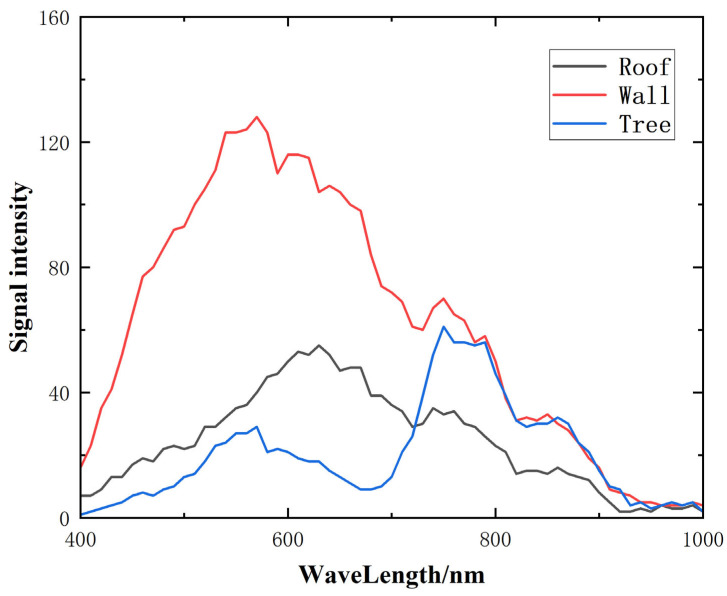
Spectral intensity curves of roofs, walls, and green trees.

The monochromatic images indicate a high level of imaging quality. In Figure 18d, the 800 nm monochrome map highlights three specific locations: the red roof, the yellow wall, and the green tree. Spectral data acquired from these positions were plotted to generate the spectral curves presented in Figure 19. The analysis reveals that the red roof exhibits a prominent peak at 630 nm, while the yellow wall shows a peak at 570 nm. Additionally, the green leaves demonstrate peaks at both 560 nm and 760 nm. The distinct characteristics of these spectral curves confirm that the designed high-resolution convex grating dispersive hyperspectral imaging system possesses excellent detection capabilities for spectral imaging.

### 2.5. Integration of Unmanned Airborne Hyperspectral Remote Sensing Detection Systems

**Figure 20:**
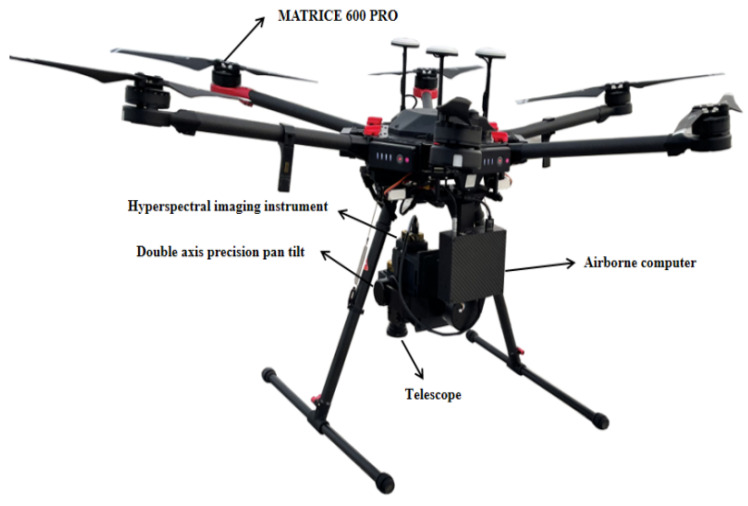
Components of an unmanned airborne hyperspectral remote sensing system.

The remote sensing equipment comprises an independently developed high-resolution convex grating dispersive hyperspectral imager system, which integrates sensing, transmission, and computing control functions. The system is primarily divided into five components: the multi-rotor UAV, the precision stabilized gimbal, the on-board computer, the hyperspectral imager, and the control and data acquisition software module, as illustrated in Figure 20. The stabilizing gimbal is securely mounted beneath the UAV, equipped with a built-in self-stabilizing algorithm that enables automatic stabilization upon powering on. The hyperspectral imager is affixed to the gimbal, with its position adjusted so that under the gimbal’s active stabilization, the objective lens of the hyperspectral camera points directly toward the ground. Furthermore, the orientation of the camera’s slit is configured to be perpendicular to the flight direction of the UAV. The microcomputer is mounted at the rear of the gimbal bracket, with both the computer and gimbal connected to the UAV’s power supply. The hyperspectral camera interfaces with the microcomputer via a data cable. The on-board computer is equipped with proprietary camera control software that facilitates the collection, storage, and processing of data.

## 3. Experiment and Results

### 3.1. Flight Experiment on an Unmanned Aircraft-Mounted Hyperspectral Remote Sensing System

**Figure 21:**
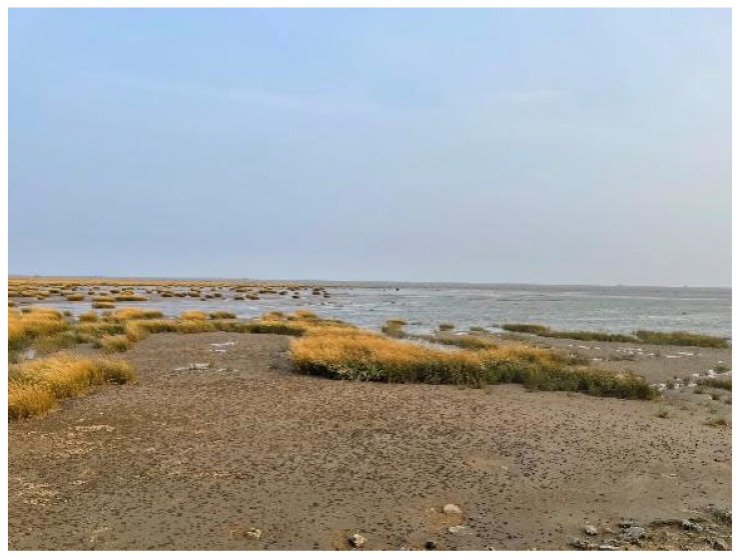
Photo of the distribution area of *Spartina alterniflora* at Yangkou Beach.

The experimental area was primarily located at Yangkou Beach in Shouguang City, Shandong Province, which is recognized as a significant distribution zone for *Spartina alterniflora* around the Bohai Sea, as depicted in Figure 21. To minimize spectral data saturation caused by solar radiation-induced flares, integration time was reduced, and data collection was scheduled between 10:00 AM and 2:00 PM. During this timeframe, the influence of atmospheric radiation could be effectively disregarded [16].

Simultaneously, preliminary preparations were conducted, including planning the route for the unmanned aerial vehicle (UAV), setting integration time, adjusting flight speed, and determining flight altitude. Utilizing intelligent analysis software for UAV data, the hyperspectral imaging instrument mounted on the UAV successfully acquired data from the distribution area of *Spartina alterniflora* at an altitude of 100 m. The entire experimental process was completed within 20 min, ensuring that the angle of solar radiation remained relatively stable, thereby minimizing any potential impact on the experimental data.

**Figure 22:**
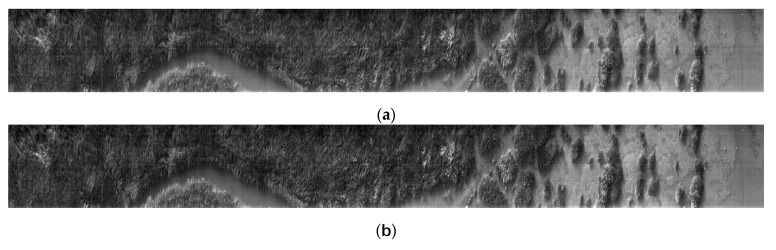

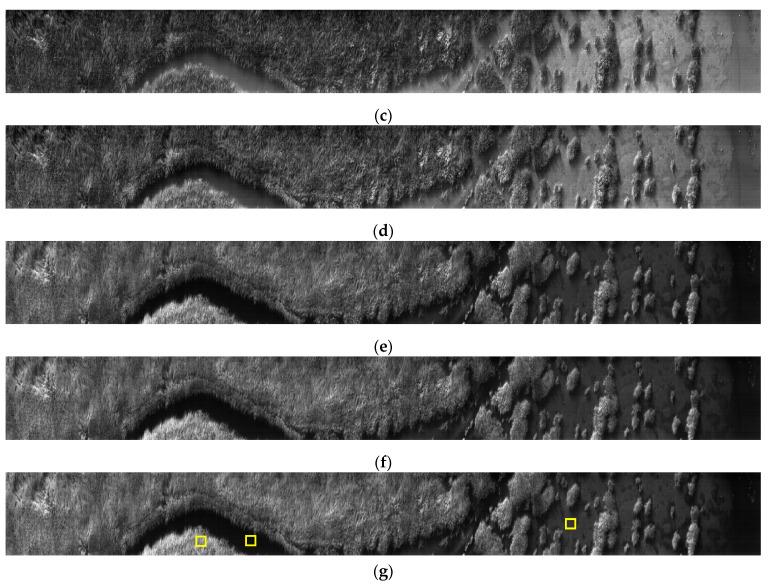
Monochromatic images of *Spartina alterniflora* captured by unmanned aerial vehicle hyperspectral system in different frequency bands: (**a**) monochromatic image in the 560 nm wavelength band; (**b**) monochromatic image in the 600 nm wavelength band; (**c**) monochromatic image in the 650 nm wavelength band; (**d**) monochromatic image in the 700 nm wavelength band; (**e**) monochromatic image in the 750 band; (**f**) monochromatic image in the 800 nm wavelength band; (**g**) monochromatic image in the 850 nm wavelength band.

**Figure 23:**
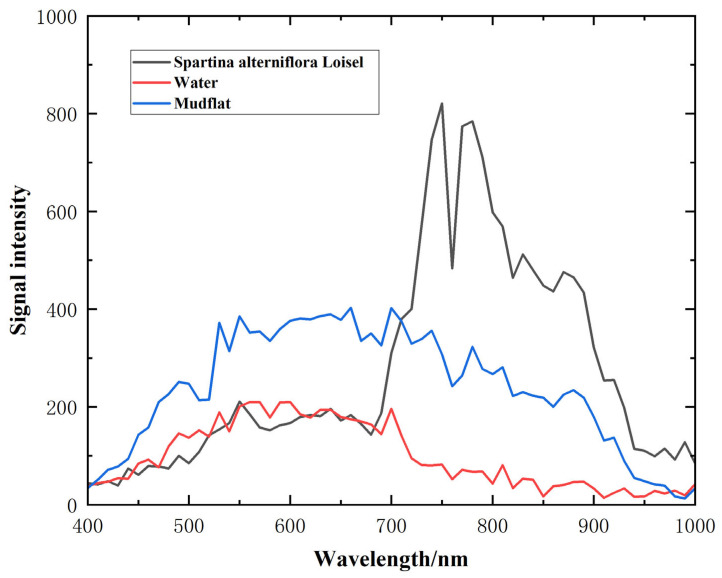
Spectral curves of *Spartina alterniflora* Loisel, water, and mudflat.

After the experiment, the preprocessed hyperspectral data were taken out for further processing and analysis. Figure 22 shows the monochromatic graphs of 570 nm, 620 nm, 670 nm, 720 nm, 770 nm, 820 nm, and 870 nm wavelengths. Figure 23 shows the spectral curves of *Spartina alterniflora*, water areas, and mudflat. The selected spectral positions are labeled in Figure 22g. From the monochromatic images, it can be seen that the flight scanning experiment results have no obvious distortion, and the imaging quality is excellent, indicating that the developed unmanned airborne hyperspectral remote sensing detection system has excellent performance

### 3.2. Classification of Hyperspectral Data of Spartina alterniflora

**Figure 24:**
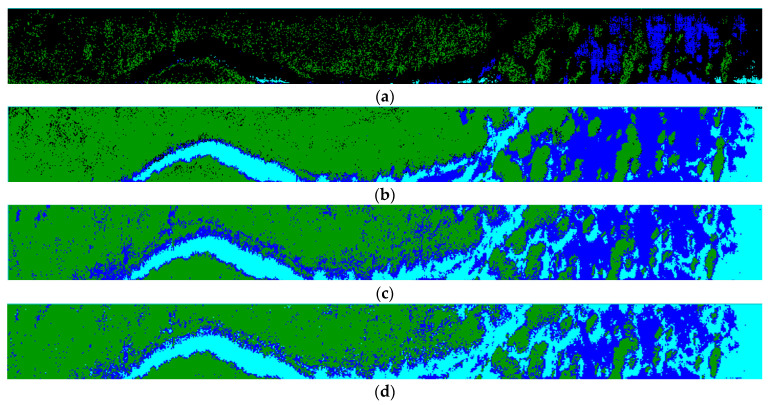
Classification results of hyperspectral data of *Spartina alterniflora* using different classification algorithms; among them, green represents *Spartina alterniflora*, light blue represents water area, and dark blue represents mudflat. (**a**) SAM classification results; (**b**) SID classification results l; (**c**) SVM classification results; (**d**) BPNN classification results.

For the hyperspectral data of *Spartina alterniflora*, different classification methods can be directly used to analyze its area proportion and distribution, providing accurate data support for formulating effective governance plans. The classification algorithms of spectral angle mapping (SAM), spectral information divergence (SID), support vector machine (SVM) and back-propagation neural network (BPNN) were used to classify *Spartina alterniflora*, waters, and mudflat in the test area. The SAM algorithm is an algorithm based on the overall similarity of spectral curves, which considers the spectrum of each pixel in an image as a high-dimensional vector. The similarity between spectra is measured by calculating the angle between the two vectors. The smaller the angle, the more similar the two spectra are, and the greater the possibility of belonging to the same class. Therefore, the category of unknown data can be distinguished based on the size of the spectral angle. The SID algorithm is a spectral classification method based on information theory to measure the difference between two spectra. The spectral vector is treated as a random variable, and the similarity between the two random vectors is analyzed based on probability and statistical theory. That is, the smaller the value of spectral information divergence, the more similar the two sets of spectra. SAM and SID are both traditional remote sensing image classification algorithms that are relatively easy to implement. The SVM algorithm is a machine learning method based on statistical learning theory. SVM can automatically search for support vectors with high discriminative ability for classification and construct classifiers based on them. It can maximize the interval between classes, thus having good generalization and high classification accuracy. The BPNN algorithm refers to the use of computers to simulate the structure of the human brain, using many small processing units to simulate biological neurons and using algorithms to achieve the recognition, memory, and thinking processes of the human brain. It has great advantages in solving complex nonlinear, uncertain, and uncertain problems. The classification results are shown in Figure 24. The classification results of SAM and SID show that there are wrong and mixed classifications, and the classification accuracy is low. The classification accuracy of the SVM and neural network classification algorithms is relatively high, which can show the distribution position of *Spartina alterniflora* in detail. As shown in the figure, *Spartina alterniflora* continues to expand to deep water.

Table 3 mainly evaluates the accuracy of different classification algorithms through the kappa coefficient and overall accuracy OA and calculates the proportion area of *Spartina alterniflora* under different algorithm conditions. The experimental results show that the SAM algorithm has low classification accuracy and reference value, while the classification accuracy of other algorithms is higher than 80%. The main reason for the high accuracy is that in areas where *Spartina alterniflora* exists, other organisms are almost extinct, and there are few classification types and significant differences in spectral characteristics of classified objects, resulting in overall high classification accuracy. Therefore, dynamic analysis of the distribution of *Spartina alterniflora* can be achieved through SVM and BPNN classification algorithms. The analysis results show that the area of *Spartina alterniflora* in the study area accounts for a relatively large proportion, exceeding half of the entire area, indicating a relatively serious degree of invasion.

## 4. Conclusions

This study successfully developed a high-resolution convex grating dispersive hyperspectral imaging system designed for unmanned aerial vehicles (UAVs). The system features a spectral range of 400 nm to 1000 nm, with a total weight of 1.5 kg and dimensions of 200 mm × 167 mm × 78 mm. It offers several advantages, including light-weight construction, compact size, a wide field of view, and high resolution. Following land scanning tests that demonstrated excellent imaging quality, the system was stabilized by a gimbal and integrated into the UAV for push-broom imaging of the Yangkou Beach area. Utilizing classification algorithms such as back-propagation neural network (BPNN) and support vector machine (SVM), we classified the hyperspectral data of *Spartina alterniflora* collected during flight experiments. This analysis yielded precise estimates of the area’s proportion of *Spartina alterniflora* within the study region. Consequently, the system facilitates intelligent monitoring of the research area with high timeliness and accuracy, providing significant reference value for conservation efforts related to *Spartina alterniflora*.

## Figures and Tables

**Table 1:** Technical specifications of imaging spectrometer.

Parameters	Values
Spectral range/nm	400–1000 nm
Field of view/(°)	30°
Focal length of telescope f′/mm	20
Diameter of entrance pupil/mm	6.1
Detector array size/pixel	2048 × 2048
Detector pixel size/μm	5.5 × 5.5
Spectral resolution/nm	≤5 nm
Modulation transfer function	≥0.5@45 lp/mm
Volume	≤200 mm × 170 mm × 80 mm
Weight	≤1.5 kg

**Table 2:** Calculated resolution of Hg light spectrum.

Characteristic Wavelength/nm	Center Element Position	FWHM	Spectral Resolution/nm
404.66	440.1796	3.08615	1.568062
546.08	718.07717	2.97023	1.509163521
696.54	1014.23	3.88458	1.973742
706.72	1034.255	3.89649	1.979793
738.4	1096.581	5.19572	2.639927
763.51	1146.329	7.25692	3.687216
772.4	1163.566	6.01151	3.054427
794.82	1207.712	5.3514	2.719028
912.3	1439.545	6.27595	3.188788

**Table 3:** Classification accuracy.

Arithmetic	Kappa Coefficient	OA/%	Area Proportion/%
SAM	0.40	51.0	30.4
SID	0.92	95.2	64.08
SVM	0.99	99.8	52.9
BPNN	1	100	53.1

## Data Availability

Data are contained within the article.
